# Metabolic Profiling of Xylooligosaccharides by Lactobacilli

**DOI:** 10.3390/polym12102387

**Published:** 2020-10-16

**Authors:** Ilia Iliev, Tonka Vasileva, Veselin Bivolarski, Albena Momchilova, Iskra Ivanova

**Affiliations:** 1 Department of Biochemistry and Microbiology, Plovdiv University, 4000 Plovdiv, Bulgaria; vasileva@uni-plovdiv.bg (T.V.); bivolarski@uni-plovdiv.bg (V.B.); 2 Centre of Technologies, Plovdiv University, 4000 Plovdiv, Bulgaria; 3 Institute of Biophysics and Biomedical Engineering, Bulgarian Academy of Sciences, 1113 Sofia, Bulgaria; albena@obzor.bio21.bas.bg; 4 Department of General and Applied Microbiology, Sofia University, 1164 Sofia, Bulgaria; iskrai3@yahoo.com

**Keywords:** xylooligosaccharide utilization, prebiotics, lactobacilli, liver membrane phospholipids

## Abstract

Three lactic acid bacteria (LAB) strains identified as *Lactobacillus plantarum*, *Lactobacillus brevis*, and *Lactobacillus sakei* isolated from meat products were tested for their ability to utilize and grow on xylooligosaccharides (XOSs). The extent of carbohydrate utilization by the studied strains was analyzed by HPLC. All three strains showed preferences for the degree of polymerization (DP). The added oligosaccharides induced the LAB to form end-products of typical mixed-acid fermentation. The utilization of XOSs by the microorganisms requires the action of three important enzymes: β-xylosidase (EC 3.2.1.37) exo-oligoxylanase (EC 3.2.1.156) and α-L-arabinofuranosidase (EC 3.2.1.55). The presence of intracellular β-D-xylosidase in *Lb. brevis*, *Lb. plantarum*, and *Lb. sakei* suggest that XOSs might be the first imported into the cell by oligosaccharide transporters, followed by their degradation to xylose. The studies on the influence of XOS intake on the lipids of rat liver plasma membranes showed that oligosaccharides display various beneficial effects for the host organism, which are probably specific for each type of prebiotic used. The utilization of different types of oligosaccharides may help to explain the ability of *Lactobacillus* strains to compete with other bacteria in the ecosystem of the human gastrointestinal tract.

## 1. Introduction

There is evidence that prebiotics can impart a range of health benefits if consumed on a regular basis. There have been a number of excellent papers and reviews on the topic of prebiotics and their health benefits [1].

Galactooligosaccharides (GOSs) and fructooligosaccharides (FOSs) have an established prebiotic status, and their health effects have been extensively studied [2,3]. There is a growing commercial interest in generating new non-digestible oligosaccharides for functional food ingredients, and a variety of other potentially prebiotic compounds already exist such as xylooligosaccharides [2,4].

Xylooligosaccharides are sugar oligomers made up of xylose units, which appear naturally in bamboo shoots, fruits, vegetables, milk, and honey. Their production at an industrial scale is carried out from lignocellulosic materials. Depending upon various xylan sources used for XOS production, the structures of XOSs vary in the degree of polymerization (DP), monomeric units, and types of linkages. Generally, XOSs are mixtures of oligosaccharides formed by xylose residues linked through β-(1→4)-linkages [5]. The number of xylose residues involved in their formation can vary from 2 to 10 and they are known as xylobiose, xylotriose, and so on. For food applications, xylobiose (DP = 2) is considered to be a xylooligosaccharide [6].

Degradation and utilization of XOSs by probiotic microorganisms began in the early works of Okazaki who studied in vitro fermentations with *Bifidobacterium adolescentis*, *Bifidobacterium longum*, and *Bifidobacterium infantis.* The degradation and utilization of XOSs are strain-specific and are also affected by the DP of oligomers present in the XOS mixture [7,8]. Later, Hopkins carried out fermentations with commercial XOSs (Suntory, Japan; 70% purity and DP = 2–4) and found that the ability of bifidobacteria to grow on XOSs depended on the considered strain [9]. The influence of oat bran oligosaccharides on carbohydrate utilization and fermentation end-products was studied with *Lactobacillus rhamnosus*, *Lactobacillus plantarum*, and *Lactobacillus lactis* [10,11]. The main products of LAB metabolism were lactic acid, acetic acid, formic acid, and ethanol. The results indicated that XOSs induce LAB to form the end-products of typical mixed-acid fermentation which was mainly due to the starvation of cells.

Efficient and complete degradation of XOSs requires the cooperation of different enzymes including β-xylosidase, α-glucuronidase, α-L-arabinosidase, and acetyl xylan esterase. Strains of *Leuconostoc lactis* SHO-47 and *Lb. lactis* SHO-54 were cultivated with a hydrolyzed birchwood xylan as the unique carbon source to produce D-lactic acid. It was suggested that the xylosidase enzyme of these two strains is localized in their cytoplasm [12].

With a growing market for prebiotic-containing foods, there is increasing interest in understanding how prebiotics function at the molecular level. Advances in the genomics of lactobacilli and bifidobacteria have enabled modeling of transport and catabolic pathways for prebiotic utilization [13]. Recently, it was shown that nine of the ten genes that encode proteins predicted to play a role in XOS catabolism in *Bifidobacterium animalis* subsp. *lactis* BB-12 (i.e., XOS-degrading and metabolizing enzymes, transport proteins, and a regulatory protein) were induced by XOSs at the transcriptional level, and the proteins encoded by three of these (β-D-xylosidase, sugar-binding protein, and xylose isomerase) showed higher abundance on XOSs. Xylooligosaccharides are degraded intracellularly through the action of xylanases and xylosidases to D-xylose, which is subsequently metabolized by the D-fructose-6-P shunt [14].

Investigations on prebiotics are related mainly to their influence on the gastrointestinal tract [15] and on the serum lipids and carbohydrates [16]. There are scarce studies dealing with the effect of prebiotics on liver lipogenesis concerning predominantly triglycerides and fatty acid re-esterification [17]. However, quite a few studies are devoted to the impact of prebiotics on liver membrane lipids, their metabolism, and susceptibility to peroxidation.

In this study, three *Lactobacillus* strains (*Lactobacillus plantarum* S26, *Lactobacillus brevis* S27, and *Lactobacillus sakei* S16) and the effect of XOS supplementation on *Lactobacillus* growth, metabolite products (end-products of fermentation), and enzyme activity involved in the utilization of XOSs were evaluated. Special attention was paid to the effect of XOSs on liver lipogenesis concerning predominantly triglycerides and fatty acid re-esterification.

## 2. Materials and Methods

### 2.1. Bacterial Strains and Culture Conditions

In this study, we used three strains of lactobacilli from the collection of the Department of General and Applied Microbiology at Sofia University (Sofia, Bulgaria): *Lactobacillus plantarum* S26, *Lactobacillus brevis* S27, and *Lactobacillus sakei* S16. The strains were cultured overnight (16–18 h) on MRS (de Mann Rogosa Sharpe broth, Merck, Darmstadt, Germany) media [18] at 37 °C and in limitation of oxygen (BBL^®^ Gas Pak anaerobic system envelopes, Becton Dickinson, Franklin Lakes, NJ, USA).

### 2.2. Carbohydrates Used in This Study

Xylooligosaccharides (purity 95%) presenting different degrees of polymerization were purchased commercially (Shandong Longlive Bio-technology Co., Ltd., Zibo, China). The concentration of the XOSs was set to 2% to an MRS broth. Glucose, xylose, and xylobiose (purity 99%, Merck, Darmstadt, Germany) were used as controls. Each carbohydrate was sterilized by filtration through a 0.2-µm sterile filter (Sartorius, Gottingen, Germany), and the pH of the solutions was not adjusted. All examinations were performed at least twice.

### 2.3. Fermentation

Lactobacilli were routinely grown in an MRS broth (Merck) [18]. Overnight grown cells were washed twice in saline (0.85% NaCl solution), and 10% of the bacterial suspension (10^7^ cfu mL^−1^) was used to inoculate modified MRS broth and agar media (pH 6.8) containing either 2% glucose, 2% xylose, 2% xylobiose, or 2% XOS. The anaerobic fermentations were performed in 100-mL glass bottles at 37 °C for 48 h (BBL^®^ Gas Pak anaerobic system envelopes, Becton Dickinson, NJ, USA).

### 2.4. Analytical Assays

#### 2.4.1. Microbial Growth

To evaluate the influence of the above-mentioned different carbon sources on the growth of the bacterial strains, anaerobic fermentations were carried out in triplicate. Bacterial growth was measured by a turbidimetric method at 600 nm and calibrated against cell dry weight using a spectrophotometer (UV/Vis Shimadzu, Kyoto, Japan). For each experiment, data were analyzed using the Excel statistical package. The optical density (OD) readings and standard deviations were calculated from duplicate samples from two separate experiments.

The growth of the studied strains was monitored in an MRS broth medium supplemented with the tested oligosaccharides, at a concentration of 2%, as a sole carbon source. A 4% pre-washed inoculum of a given strain overnight culture (1 × 10^9^ cfu mL^−1^) was used to examine the effect of the pre-determined oligosaccharides on the growth of the studied strains. Cultures (200 mL per cultivation) were incubated at 37 °C for a period of 48 h under non-pH-controlled conditions. The growth of each strain was monitored by measuring the OD of the cultures at 0, 6, 12, 24, and 48 h at 600 nm.

#### 2.4.2. Analysis of Metabolites

Lactic acid was determined enzymatically with L-lactate dehydrogenase and D-lactate dehydrogenase (commercially available kit 10 139 084 035, Boehringer Mannheim, Mannheim, Germany) according to the instructions of the manufacturer.

Acetic acid was determined enzymatically with acetyl-CoA synthetase, citrate synthase, malate dehydrogenase (commercially available kit 10 148 261 035, Boehringer Mannheim, Mannheim, Germany) according to the instructions of the manufacturer.

Ethanol was determined enzymatically with alcohol dehydrogenase and aldehyde dehydrogenase (commercially available kit 10 176 290 035, Boehringer Mannheim, Mannheim, Germany) according to the instructions of the manufacturer.

All the analyses for the determination of lactic acid, acetic acid, and ethanol were performed in duplicate from different experiments.

#### 2.4.3. Analysis of Carbohydrates and Proteins

The oligosaccharides were analyzed by HPLC using a Symmetry C18 column (4.6 mm× 150 mm) and a Waters 1525 Binary HPLC Pump (Waters, Milford, MA, USA). Oligosaccharides were detected by using a Waters 2414 refractive index detector. The products were identified in the chromatograms as described by Remaud-Simeon and co-workers [19].

Residual sugars (glucose, xylose, and XOSs in the fermentation broth after fermentation) were determined by HPLC using a Zorbax carbohydrate column (4.6 mm × 150 mm; Agilent, Santa Clara, CA, USA), analytical guard column Zorbax NH2 (4.6 mm× 12.5 mm), and a mobile phase of 75/25 acetonitrile/water. Breeze Chromatography Manager Software (Waters) (v2, Waters, Milford, MA, USA) was used for data treatment.

Proteins were assayed by the method of Lowry [20].

All the analyses for the determination of sugars and proteins were performed in duplicate from different experiments.

#### 2.4.4. Isolation of Liver Plasma Membranes

Plasma membranes from rat livers were isolated according to a procedure involving liver homogenization and differential centrifugation. Briefly, the post-nuclear supernatant was loaded on a discontinuous sucrose gradient and centrifuged at 100,000× g for 2.5 h. The plasma membrane fraction was obtained at a density of 8% (*w/v*), suspended in ice-cold 10 mM Tris buffer, pH 7.4, and used immediately for lipid analysis.

#### 2.4.5. Lipid Extraction and Analysis

Lipid extraction was performed with chloroform/methanol (2:1, *v/v*). The organic phase obtained after extraction was concentrated and analyzed by thin-layer chromatography. The phospholipid fractions were separated on silica gel G 60 plates in a solvent system containing chloroform/methanol/2-propanol/triethylamine/0.25% KCl (30:9:25:18:6, *v/v*). The location of the separate fractions was determined by spraying the plates with 2′,7′-dichlorofluorescein. The spots were scraped and quantified by the determination of the inorganic phosphorus. Cholesterol content was assayed by gas chromatography using a medium polarity RTX-65 capillary column (0.32 mm internal diameter, length 30 m, thickness 0.25 µm). Calibration was achieved by a weighted standard of cholestane.

#### 2.4.6. Fatty Acid and Phospholipase A2 Analysis

The phospholipid extracts were saponified with 0.5 N methanolic KOH and methylated with boron trifluoride-methanol complex (Merck). The fatty acid methyl esters were extracted with hexane and separated by gas chromatography on a capillary column coated with Supelcowax 10-bound phase 9 (i.d. 0.32 mm, length 30 m, film thickness 0.25 µm, Supelco, Bellafonte, PA, USA) fitted in a Perichrom gas chromatograph (Perichrom, Saulx-les-Chartreux, France). Phospholipase A2 activity was assayed by quantification of the fatty acid products by gas chromatography.

All the analyses for the determination of phospholipase A2 activity were performed in duplicate from different experiments.

#### 2.4.7. Determination of Lipid Peroxidation

The lipid peroxidation was assessed by measuring the loss of cis-parinaric acid (PNA) fluorescence. The hepatocyte plasma membranes were incubated with 10 μM PNA at 37 °C for 30 min. The buffer was removed, and the plasma membranes were washed 3 times with warm PBS to remove the unincorporated dye. The membranes were transferred to test tubes with 2 mL 10 mM Tris buffer, pH 7.4, and fluorescence was measured at 502 nm (excitation beam) and 520 nm (emission beam). The level of lipid peroxidation was determined as fluorescence intensity per mg of protein.

All the analyses for the determination of lipid peroxidation were performed in duplicate from different experiments.

#### 2.4.8. Enzymatic Activity

The bacteria cells were washed twice with 50 mM sodium acetate buffer (pH 6.5) and suspended in 1 mL lysis buffer (50 mM sodium acetate buffer pH 7.5, 300 mM sodium chloride, and 2 % glycerol). The sonication of bacteria cells was performed with a UP 50 H Ultrasonic Processor (Hielscher Ultrasound Technology, Teltow, Germany) for 15 cycles and 50 % amplitude. Each cycle had a duration of 5 s and 2 min break between cycles on ice. After sonication, the lysates were centrifuged (12,000 rpm, 10 min, 4 °C), and the supernatants were collected for evaluation of α-glucosidase, α-galactosidase, and β-xylosidase activity in lactobacilli strains.

β-glucosidase activity was measured according to the procedure described by Dewi and others. [21,22]. The amount of p-nitrophenol (pNP) released by the degradation of the substrate pNP-β-D-glucopyranoside (Sigma-Aldrich, St. Louis, MI, USA) was estimated. Briefly, to 100 μL of the bacterial lysate, we added 250 μL of 5 mM pNP-α-D-glucopyranoside (pH 6.8) and 150 μL water and then incubated it for 10 min at 37 °C. The enzyme process was stopped by adding 2 mL 1 M Na_2_CO_3_. Finally, the amount of pNP was determined by measuring the absorbance at 405 nm.

β-xylosidase activity was estimated by the method of Lasrado and Gudipati [22] as the amount of pNP released by the substrate degradation of pNP-β-D-xylopyranoside (Sigma-Aldrich, St. Louis, MI, USA). To 100 μL of the bacterial lysate, we added 900 μL 5 mM pNP-β-D-xylopyranoside (pH 5.7) and 100 μL water. The mixture was incubated for 30 min at 30 °C. The enzyme process was stopped by adding 100 μL saturated sodium tetraborate solution. The amount of pNP was quantified at 405 nm.

### 2.5. Statistical Analysis

Programmable scientific calculator “CASIO” fx-991ES Plus, statistical software package SigmaPlot v12.0 (Systat Software, Inc., Chicago, IL, USA), and Microsoft Excel were used for data analysis and graphical representation.

## 3. Results

The results of the performed screening procedure of *Lactobacillus* strains (*Lb. plantarum*—13; *Lb. brevis*—2; *Lb. sakei*—1) show that the examined strains fermented XOSs with different capacity, changing the optical density [23]. Our next investigation was focused on the properties of three of the strains: *Lb. plantarum* S26, *Lb. brevis* S27, and *Lb. sakei* S16.

### 3.1. Changes in the Enzyme Activities

The growth and production of lactic acid and acetic acid of the three bacterial *Lactobacillus* strains in a modified MRS broth supplemented with XOSs are shown in Table 1. Growth was evaluated in terms of maximum optical density at 600 nm and the concentration of lactic acid and acetic acid achieved during 48 h fermentation. Kinetic growth on glucose was used as control. All studied *Lactobacillus* strains fermented XOSs in a different manner.

In the current study, the activity of β-xylosidase and β-glucosidase was detected during cultivation on XOSs. The results are shown in Table 2. No activity of the mentioned enzymes in the three strains grown on glucose media was detected. The highest β-xylosidase activity was shown for *Lb. brevis* S27 at 48 h of cultivation. On the other hand, the activity of xylanase was detected for *Lb. brevis* S27 and *Lb. sakei* S16 but not for *Lb. plantarum* S26. In the case of β-glucosidase, the result shows that this activity appears during the cultivation on XOS media.

β-Xylosidase is an intracellular enzyme. As shown in Table 3, β-xylosidase activity was not found when the studied strains were cultivated in the presence of xylose. On the other hand, β-xylosidase activity was not found in the supernatant of the cultures in contrast to the cell-free extract of the three strains (Table 4). Then, β-xylosidase activities in the cytoplasm and in the membranes were assayed. The activity was observed only in the cytoplasm (Table 4). To verify that resting cells release β-xylosidase during incubation, a cell suspension was centrifuged, and 4-nitrophenil β-D-xylopiranoside (PNPX) was added to the supernatant before and after incubation. Production of 4-nitrophenol was not observed, which means that β-xylosidase was not released from the resting cells by cell lysis.

Xylobiose is a better inducer of β-xylosidase than XOSs. The next studies were performed to prove the induction of xylosidase in the presence of xylose, xylobiose, and XOSs. In the studied strains, β-xylosidase activity was induced by xylobiose and XOS but not by xylose (Table 3). From the presented results it is clear that the activity of β-xylosidase is 60% higher in the presence of xylobiose in comparison with XOSs. In the presence of xylobiose, the concentration of xylose increased in the resting-cells suspension for the studied strains, whereas its concentration decreased in the presence of xylotriose and XOSs (Table 5).

### 3.2. Analysis of the Residual Xylooligosaccharides

**Figure 1:**
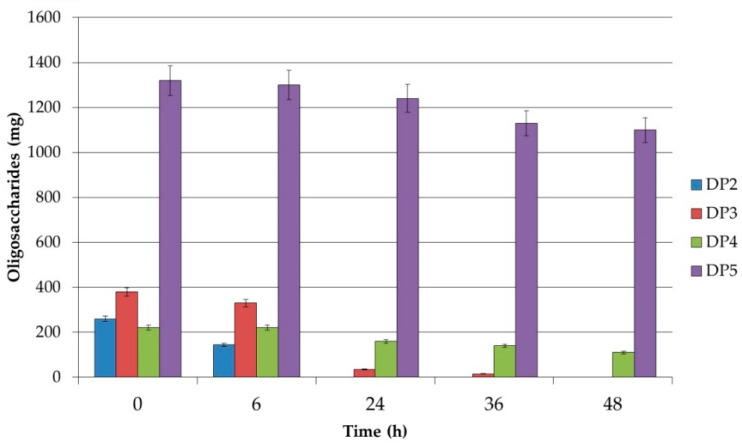
Residual amounts of oligosaccharides during the fermentation of XOSs by *Lb. plantarum* S26. DP: degree of polymerization.

**Figure 2:**
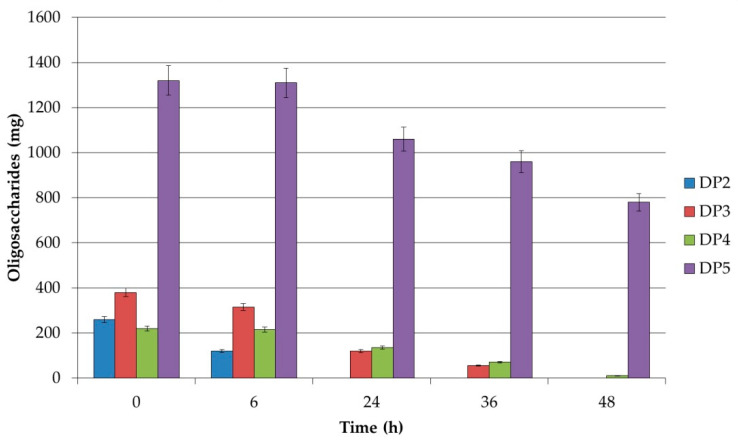
Residual amounts of oligosaccharides during the fermentation of XOSs by *Lb. brevis* S27.

**Figure 3:**
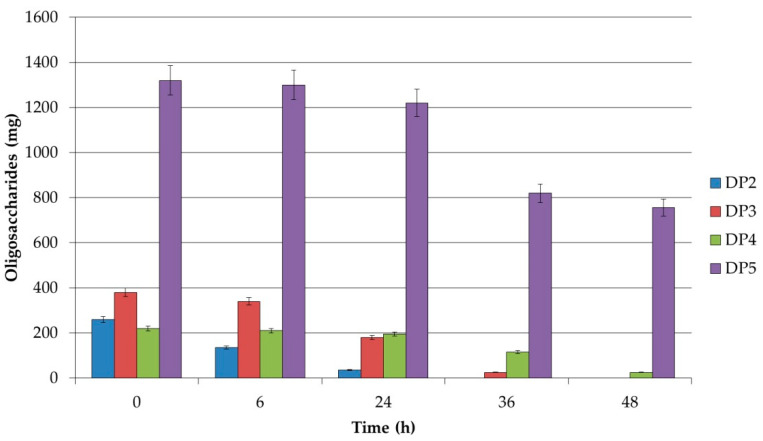
Residual amounts of oligosaccharides during the fermentation of XOSs by *Lb. sakei* S16.

The extent of carbohydrate utilization by the studied strains *Lb. plantarum* S26, *Lb. brevis S27*, and *Lb. sakei* S16 was analyzed by HPLC. All three strains showed preferences for the degree of polymerization (DP). From the received results it is clear that the short-chain XOSs with a DP up to 5 were utilized most intensively, and practically no utilization was confirmed for a DP higher than 5. It is interesting that strain *Lb. plantarum* S26 utilized xylobiose during the first 24 h of cultivation and 96% of xylotriose until 36 h (Figure 1). It could be noted that only 14% of XOSs with DP = 5 were utilized after 36 h of cultivation. Strain *Lb. brevis* S27 utilized xylobiose and xylotriose in the same manner as *Lb. plantarum* S26, but XOSs with DP = 5 were used by the cells after 24 h of growth until 41% of the total quantity. *Lb. sakei* S16 utilized XOSs in the same way as *Lb. brevis* S27 (Figure 2 and Figure 3).

In these strains, the uptake rate of xylobiose, utilization of XOSs, and the hydrolytic rate by xylosidase in the cell were higher than the metabolic rate of xylose which is excreted out of the cells. This supports the hypothesis that in the resting cell only the hydrolysis of xylobiose, xylotriose, and XOSs was stimulated, but the obtained xylose was not metabolized. These results confirm the data obtained by HPLC analysis. The utilization of XOSs was found to be strain-specific. For the future application of XOSs as prebiotics, it is recommended to use strains with different potential of the degradation of xylobiose, xylotriose, and XOSs with a DP of more than 5.

### 3.3. Production of Lactic Acid, Acetic Acid, and Ethanol

The fermentation pattern depends on the physiological conditions of the growing cells. When cultivated on XOSs, the studied strains produced different amounts of acetic and lactic acid (Table 6). The fermentation end-products were measured after cultivation of the strains in media with XOSs or xylobiose as growth substrates, and it was observed that the production of lactic acid and acetic acid was higher in all cases when xylobiose was used. The ratio of lactic/acetic acid was determined to be from 2:0.1 to 2:1 after 48 h of cultivation when the main carbon source was glucose. When the main carbon source was xylobiose, the ratio of lactic/acetic acid was changed by different strains from 1:1 (strain S26) to 1:1.3 (strains S16 and S27). The ratio was dramatically changed to 1:1.5 (strain S26) and 1:2 (strain S27) when the main carbon source was XOSs. After comparing the data on the amount of lactic acid and acetic acid and the evaluation of the dynamics during the cultivation process, the established ratios are observed at the end of the process.

### 3.4. Influence of XOSs on Liver Membrane Lipids, Their Metabolism, and Susceptibility to Peroxidation

**Figure 4:**
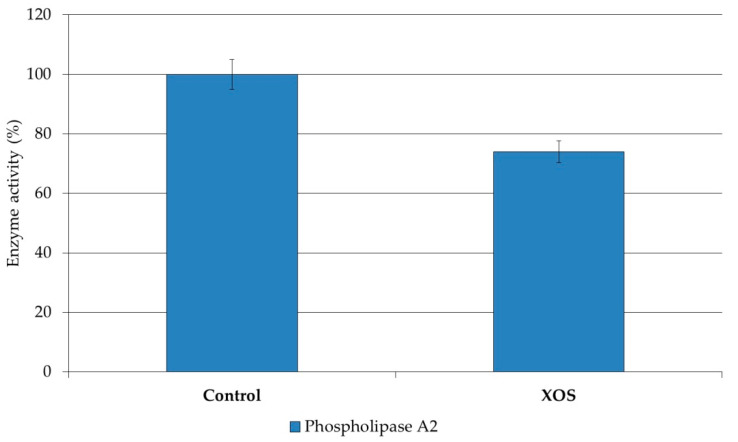
Alterations in the activity of PLA2 in liver plasma membranes isolated from XOS-fed rats.

Investigations on prebiotics are related mainly to their influence on the gastrointestinal tract [15] and on serum lipids and carbohydrates [16]. There are scarce studies dealing with the effect of prebiotics on liver lipogenesis concerning predominantly triglycerides and fatty acid re-esterification [17]. However, quite a few studies are devoted to the impact of prebiotics on liver membrane lipids, their metabolism, and susceptibility to peroxidation. In our previous studies, we reported alterations in liver membrane lipid composition and structural organization as a result of the intake of fructooligosaccharides [24]. In the present study, we analyzed the impact of XOS intake on the lipid composition and susceptibility to the oxidative attack of cholesterol, the latter being altered in the liver plasma membranes of XOS-fed rats. The obtained results show that the content of phosphatidylcholine (PC) was increased and that of lysophosphatidylcholine (LPC) was reduced as a result of XOS intake (Table 7). To elucidate the biochemical mechanism responsible for the observed changes in the liver plasma membrane lipids, we determined the activity of membrane-bound phospholipase A2 (PLA2), which is responsible for the conversion of PC into LPC (Figure 4). As evident from Figure 4, this enzyme activity was reduced as a result of XOS intake. Thus, one possible reason for the changes in the liver plasma membrane lipids could be the observed decrease of membrane-bound PLA2 activity. In addition, the analysis of the fatty acid composition showed a slight increase of arachidonic acid content (Table 8), which is hydrolyzed from the phospholipid molecules by PLA2.

**Figure 5:**
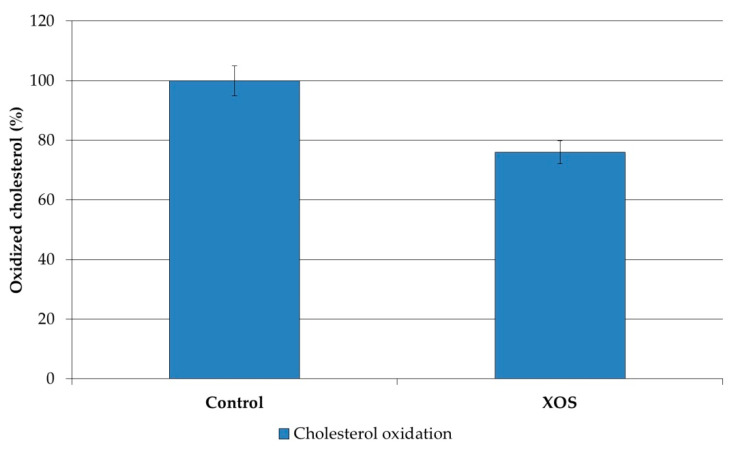
Sensitivity of membrane cholesterol to oxidative destruction in liver plasma membranes from XOS-fed rats.

**Figure 6:**
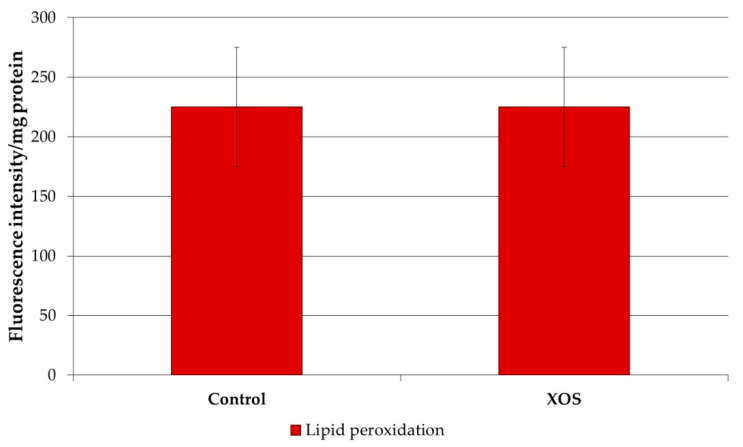
Level of lipid peroxides in the liver plasma membranes of XOS-fed rats.

On the basis of the observed alteration in the relative content of cholesterol (CH) induced by XOS intake, we analyzed the changes in the susceptibility of CH to oxidative attack. Such changes are possible because the alterations in the CH/sphingomyelin ratio lead to rearrangements of the membrane structure which often change the oxidizability of CH. The results show that XOS intake indeed reduced the susceptibility of cholesterol molecules to oxidative damage (Figure 5). Accordingly, in our previous studies on the effect of FOS intake, we reported a similar effect in liver plasma membranes. Thus, it could be summarized that oligosaccharides preserve liver membrane cholesterol against oxidative damage. However, the effect of FOSs and XOSs did not have a similar effect on the lipid peroxides in rat liver plasma membranes. The presented results demonstrate that XOS intake did not alter the content of lipid peroxides in the plasma membranes (Figure 6).

## 4. Discussion

Xylooligosaccharides (XOS) are plant-derived oligosaccharides with β-1,4 linkages between xylose (pentose) monomers that can be decorated with residues of the pentose arabinose. These residues can be linked by α-1,2 or α-1,3 bonds to xylose molecules along the chain (arabinoxylan oligosaccharides) where one or two arabinose residues can be found per xylose [25,26]. The prebiotic properties of XOSs are of great interest as functional and feed ingredients. While the XOS metabolism of *Bifidobacteriaceae* has been extensively studied, information regarding lactic acid bacteria is still limited in this context. This study provides an insight into the role of enzymes in XOS metabolism. The utilization of XOSs by the microorganisms generally requires the action of three important enzymes: β-xylosidases (EC 3.2.1.37), exo-oligoxylanases (EC 3.2.1.156), and α-L-arabinofuranosidases (EC 3.2.1.55). The presence of intracellular β-D-xylosidase in *Lb. brevis*, *Lb. plantarum*, and *Lb. sakei* suggests that XOSs might be the first imported into the cell by oligosaccharide transporters, followed by their degradation to xylose. Our previous studies and other publications are envisaged to understand in detail the metabolism of XOSs by *Lactobacillus* spp. [27,28]

Recently, it was shown that *Lb. brevis* is at least genetically equipped with functional enzymes in order to hydrolyze the depolymerization products of xylans [29]. The scientific literature available so far suggests that the ability to metabolize XOSs in in vitro assays may be a rare trait among LAB [13,30]. However, this knowledge is unfortunate, as LAB can be part of the beneficial intestinal microbiota as suggested by [31]. So far, *Lb. brevis* is the only *Lactobacillus* species reported to efficiently utilize XOSs [27,30,32,33], and an XOS-hydrolyzing β-xylosidase was recently isolated from *Lb. brevis* after the strain was grown on XOSs [34]. This is supported by the characterization of multiple ATP-binding cassette transporters (ABC) for XOSs and XOS-degrading enzymes in the species of *Bifidobacterium* [35]. In vitro growth assays and human trials that explored changes in gut microbiota composition after XOS intake also pointed to XOSs as prebiotic polysaccharides that stimulate the growth of certain lactobacilli [26]. Notwithstanding, the capacity to ferment XOSs by lactobacilli seems to be limited [23,36].

The GH43 enzymes from *Lb. brevis* DSM 20054, annotated as β-xylosidases, have been thoroughly characterized [29]. The degradation of other hemicelluloses such as xyloglucan (a β-1,4 glucan backbone with xylose residues linked to glucose via α-1,6 bonds) by lactobacilli has received less attention. *Lactobacillus pentosus* MD353, isolated from cucumber fermentation, carries a xylose operon (xylAB) involved in the metabolism of this pentose and encoding a xylose isomerase (xylA) and xylulose kinase (xylB) [37]. These enzymes convert cytoplasmic D-xylose to D-xylulose-5-phosphate, an intermediate of the pentose phosphate pathway. These species belong to the group of heterofermentative lactobacilli, thus it has been postulated that the capacity to degrade XOSs and arabinoxylans is restricted to this particular group within lactobacilli [29].

*Lb. brevis*, *Lb. plantarum*, and *Lb. sakei* are usually classified as obligatory heterofermentative. Evidence for homofermentative hexose metabolism has been presented, and it may be facultatively heterofermentative [32,38].

Previous studies concerned with in vitro growth on XOSs found little evidence for utilization in the genus *Lactobacillus. Lactobacillus fermentum* [27,30] and *Lactobacillus acidophilus* [13] were found to have moderate growth on XOSs. Chapla and co-workers [39] reported that the growth of *Lb. fermentum* and acidophilus was low compared to the growth of *Bifidobacterium* spp. The intestinal *Lactobacillus paracasei* strain was reported to be enriched by XOSs [29]. Evidence for XOS utilization by *L. lactis* [12] and *Weissella* spp. [40] has been previously reported.

We reported XOS utilization by *Lactobacillus* strains isolated from Bulgarian home artesian meat products [41]. About 30 strains identified as *Lb. plantarum*, *Lb. casei, Lb. brevis*, and *Lb. sakei* were screened for XOS utilization with the possible participation of three enzymes β-xylosidase, exo-oligoxylanase, and α-L-arabinofuranosidase alteration in the end-products and morphology of the strains cultivated in the presence of XOSs. The most promising was strain *Lb. sakei*. The effect of XOSs was studied on eukaryote membranes.

*Lb. sakei* is an important food-associated lactic acid bacterium commonly used as a starter culture for industrial meat fermentation, and potential as a biopreservative in meat and fish products. *Lb. sakei* was isolated from specific Bulgarian sausages. Previous analysis [41] showed that this strain possesses antimicrobial activity. Semi-quantitative analysis of the expression of genes involved in XOS metabolism indicated for the first time that *Lb. sakei* utilizes XOSs [42].

The main enzymes involved in the utilization of XOSs are β-xylosidase and xylanase. Research of gene expression of two enzymes involved in the degradation of XOS xylanase and β-xylosidase was performed (*Lb. plantarum* S1, S20 and S40, *Lb. brevis* S27).

*Lb. brevis* is involved in the natural fermentation of hemicellulose-rich plant food/feed (e.g., cabbage, sourdough, and forage silage), and despite the aspects mentioned below, it is also an important starter organism for the “over-attenuation” process used to produce several beer varieties [43,44,45]. However, next to *Pediococcus* spp., obligately heterofermentative *Lactobacillus* spp. (listed in group 3 according to reference 44) are commonly recognized in food spoilage, with *Lb. brevis* being one of the organisms primarily associated with wine and beer spoilage [44]. Therefore, it seems to bear a certain irony that, in the context of the enzymatic release of attractive wine aroma compounds from glycosylated precursors, we previously identified *Lb. brevis* as a versatile source of glycosidases [46,47,48].

This correlates with the fact that sugar import and phosphorylation by phosphotransferase system (PTS) are associated with homofermentative metabolism (EMP) whereas PTS components are not known to be directly involved in heterofermentative carbohydrate metabolism (phosphoketolase (PK) pathway), as phosphoenolpyruvate (PEP) does not occur as an intermediate in the PK pathway [38]. However, evidence that mannose-specific PTS transporters (EIIB) facilitate xylose uptake in several group 2 members was previously presented [39].

These results indicate that the used oligosaccharides induce LAB to form end-products of typical mixed-acid fermentation. Similar observations were proposed by Kontula and co-workers [10] when β-glucooligosaccharides and xylooligosaccharides were used as fermentative substrates and by Sjöberg [49] who observed that in a homofermentative *Lactococcus lactis* strain metabolism was altered to mixed-acid fermentation by maltose. The mechanism inducing the metabolic shift to mixed-acid fermentation has not been explained in detail, although the shift can be identified by gene expression [50]. Starvation has been suggested as one of the factors changing the metabolism from homofermentative to mixed-acid production [51,52]. Data obtained in the present study suggest that strains of LAB are individually able to change fermentation patterns, depending on the available substrates and metabolic pathways of the strains. In the colon, both monosaccharides and oligosaccharides are present, which means that both fermentation routes are possible. Human gut microbiota (HGM) is a microbial complex where dynamic mutualistic interactions related to digestion and absorption of dietary components take place. The consumption of specific food ingredients, such as prebiotics and dietary fibers, constituted mainly by carbohydrate polymers, can modulate the HGM composition and metabolism serving as a fermentable substrate to produce bacterial metabolites with beneficial effects on host health. Specifically, bacterial short-chain fatty acids, tryptophan, and organic acids have shown positive effects on pathogenic bacteria control, mineral absorption, weight control, and obesity, immune response homeostasis, gut barrier improvement, brain modulation, and anticancer activity. Despite the fact that these effects vary between individuals due to personal HGM richness, the information presented in this review contributes to understanding the effects of prebiotics and dietary fiber consumption on the generation of HGM metabolites and the mechanisms by which these metabolites interact with host cells, improving host health [53].

Our studies on the influence of XOS intake on the lipids of rat liver plasma membranes showed that oligosaccharides display various beneficial effects for the host organism, which are probably specific for each type of prebiotic used. For example, the decrease of PLA2 activity could be beneficial because the accumulation of free arachidonic acid, which is a product of PLA2 activity, is a prerequisite for the development of inflammatory processes since this fatty acid acts as a precursor for pro-inflammatory mediators, thromboxanes, and leucotriens. In addition, the reduced susceptibility of membrane cholesterol to oxidative damage is one significant effect of XOS intake because cholesterol oxidative destruction induces the accumulation of peroxide molecules and disturbs the structure and functions of membranes. At present, more profound investigations are necessary for understanding in detail the molecular mechanisms underlying the favorable effect of each separate type of oligosaccharides.

## 5. Conclusions

The impact of plant-derived XOSs on human microbiota depends on the complex relationship between their chemical structures, bacteria strain specificity, and metabolism. The ability of XOSs with a different DP to stimulate the growth of some heterofermentative *Lactobacillus* strains was proven. The investigated probiotic bacteria utilized XOS fractions, producing lactic acid, acetic acid, and ethanol as end metabolic products in different ratios. The obtained high amount of acetate and the established strain specificity of β-xylosidase and β-glucosidase enzymes induction are a prerequisite for an in-depth study of the prebiotic capacity of XOSs. Furthermore, substantial in vitro and in vivo investigations on the correlation between the probiotic properties of lactobacilli and the prebiotic activity of XOSs with different chemical structures could reveal their potential application as functional food with symbiotic characteristics.

## Figures and Tables

**Table 1:** Growth characteristics after 48 h of cultivation of different *Lactobacillus* strains on glucose, xylose, and xylooligosaccharide (XOS) media.

Strain	Carbon Source (g L^−1^)	pH (48 h)	Lactic Acid (mg mL^−1^)	Acetic Acid (mg mL^−1^)
*Lactobacillus plantarum* S26	Xylobiose	5.20 ± 0.05	1.26 ± 0.04	0.64 ± 0.01
	XOS	5.60 ± 0.07	0.84 ± 0.06	0.48 ± 0.01
	Xylose	4.80 ± 0.04	1.86 ± 0.09	0.94 ± 0.02
	Glucose	4.10 ± 0.05	2.44 ± 0.10	1.32 ± 0.02
*Lactobacillus brevis* S27	Xylobiose	4.80 ± 0.10	1.74 ± 0.08	0.84 ± 0.01
	XOS	5.10 ± 0.08	1.62 ± 0.11	0.72 ± 0.03
	Xylose	4.60 ± 0.07	2.11 ± 0.04	1.32 ± 0.03
	Glucose	3.80 ± 0.10	3.54 ± 0.02	1.87 ± 0.02
*Lactobacillus sakei* S16	Xylobiose	5.30 ± 0.05	1.23 ± 0.08	0.87 ± 0.04
	XOS	5.10 ± 0.08	0.93 ± 0.04	0.62 ± 0.06
	Xylose	4.70 ± 0.08	1,52 ± 0.03	0.95 ± 0.03
	Glucose	4.10 ± 0.07	2.37 ± 0.10	1.11 ± 0.04

**Table 2:** Changes in the activity of enzymes involved in the metabolism of xylooligosaccharides by strains of the genus *Lactobacillus.*

Enzymes	*Lb. plantarum* S26		*Lb. brevis* S27		*Lb. sakei* S26	
	Glucose	XOS	Glucose	XOS	Glucose	XOS
β-Xylosidase (U mg^−1^)	0	0.265 ± 0.15	0	1.048 ± 0.17	0	0.675 ± 0.11
β-Glucosidase (U mg^−1)^	0	0.545 ± 0.12	0	0.855 ± 0.14	0	0.219 ± 0.15

**Table 3:** Induction of β-xylosidase activity of *Lactobacillus* strains by different sugars as carbon substrates.

Strain	β-Xylosidase Activity in the Cell-Free Extracts (U mg^−1^)		
	Xylose	Xylobiose	XOS
*Lb. plantarum* S26	0	0.375 ± 0.17	0.265 ± 0.12
*Lb. brevis* S27	0	1.684 ± 0.14	1.048 ± 0.12
*Lb. sakei* S16	0	0.846 ± 0.15	0.675 ± 0.15

**Table 4:** Content of xylanase and β-xylosidase in the membranes and cytoplasm of the studied strains of the genus *Lactobacillus*, cultivated on XOS-containing media.

Strain			β-Xylosidase (%)
	Supernatant	Cytoplasm Fraction	Membrane Fraction
*Lb. plantarum* S26	0	94.6	5.4
*Lb. brevis* S27	0	95.2	4.8
*Lb. sakei* S16	0	93.6	6.4

**Table 5:** Xylose concentration after the hydrolysis of xylobiose, xylotriose, and XOSs by resting cells.

Strain	Time (min)	Xylose Concentration (g L^−1^)		
		Xylobiose	Xylotriose	XOS
*Lb. plantarum* S26	0	0	0	0
	30	0.4 ± 0.07	0.3 ± 0.11	0.3 ± 0.10
	60	0.9 ± 0.05	0.8 ± 0.05	0.6 ± 0.03
*Lb. brevis* S27	0	0	0	0
	30	0.7 ± 0.07	0.7 ± 0,05	0.5 ± 0,01
	60	1.3 ± 0.05	1.4 ± 0,05	1.1 ± 0,04
*Lb. sakei* S16	0	0	0	0
	30	0.5 ± 0.11	0.3 ± 0.05	0.3 ± 0.04
	60	1.1 ± 0.05	1.0 ± 0.04	0.8 ± 0.06

**Table 6:** Metabolic alterations by the cultivation of *Lb. plantarum* S26, *Lb. sakei* S16, and *Lb. brevis* S27 in media with XOSs.

Carbon Source	Strain	Time (h)	pH	Lactate (g L^−1^)			Acetate (g L^−1^)	Ethanol (g L^−1^)
				D-Lactate	L-Lactate	Total		
Glucose	S26	0	6.54 ± 0.03	0	0	0	0	0
		6	4.20 ± 0.05	0.98 ± 0.09	1.44 ± 0.04	2.43 ± 0.13	0.09 ± 0.01	0.08 ± 0.012
		12	4.10 ± 0.05	1.09 ± 0.09	1.54 ± 0.04	2.63 ± 0.13	0.11 ± 0.01	0.09 ± 0.012
		24	4.06 ± 0.05	1.25 ± 0.04	1.88 ± 0.03	3.13 ± 0.07	0.11 ± 0.01	0.14 ± 0.006
		48	4.02 ± 0.05	1.35 ± 0.04	1.90 ± 0.03	3.25 ± 0.07	0.15 ± 0.01	0.14 ± 0.006
	S16	0	6.54 ± 0.03	0	0	0	0	0
		6	4.35 ± 0.04	2.30 ± 0.09	1.98 ± 0.03	4.29 ± 0.12	0.21 ± 0.02	0.05 ± 0.010
		12	4.25 ± 0.04	2.37 ± 0.09	2.06 ± 0.03	4.43 ± 0.12	0.22 ± 0.02	0.05 ± 0.010
		24	4.12 ± 0.05	4.15 ± 0.16	2.04 ± 0.07	6.19 ± 0.23	0.26 ± 0.02	0.10 ± 0.012
		48	4.02 ± 0.05	4.35 ± 0.16	2.10 ± 0.07	6.45 ± 0.23	0.32 ± 0.02	0.16 ± 0.012
	S27	0	6.54 ± 0.03	0	0	0	0	0
		6	4.12 ± 0.05	2.86 ± 0.06	2.64 ± 0.04	5.50 ± 0.10	0.12 ± 0.01	0.07 ± 0.010
		12	4.00 ± 0.05	3.06 ± 0.06	2.84 ± 0.04	5.90 ± 0.10	0.16 ± 0.01	0.09 ± 0.010
		24	3.84 ± 0.04	3.84 ± 0.12	3.32 ± 0.08	7.17 ± 0.20	0.19 ± 0.02	0.10 ± 0.012
		48	3.62 ± 0.04	4.14 ± 0.12	3.62 ± 0.08	7.76 ± 0.20	0.24 ± 0.02	0.16 ± 0.012
Xylobiose	S26	0	6.55 ± 0.04	0	0	0	0	0
		6	5.10 ± 0.02	1.22 ± 0.05	0.70 ± 0.01	1.92 ± 0.06	1.59 ± 0.08	0.03 ± 0.010
		12	4.71 ± 0.03	1.79 ± 0.03	0.94 ± 0.02	2.73 ± 0.05	2.87 ± 0.09	0.06 ± 0.006
		24	4.34 ± 0.02	1.89 ± 0.03	1.04 ± 0.02	2.93 ± 0.05	2.91 ± 0.09	0.07 ± 0.006
		48	4.14 ± 0.02	1.96 ± 0.03	1.21 ± 0.02	3.17 ± 0.05	2.98 ± 0.09	0.07 ± 0.006
	S16	0	6.55 ± 0.02	0	0	0	0	0
		6	5.13 ± 0.01	0.94 ± 0.02	1.16 ± 0.05	2.10 ± 0.07	2.58 ± 0.04	0.04 ± 0.006
		12	4.81 ± 0.02	1.24 ± 0.02	1.31 ± 0.05	2.55 ± 0.07	2.88 ± 0.04	0.06 ± 0.006
		24	4.72 ± 0.02	1.33 ± 0.03	1.37 ± 0.06	2.70 ± 0.09	3.25 ± 0.03	0.07 ± 0.010
		48	4.53± 0.02	1.42 ± 0.03	1.38 ± 0.06	2.80 ± 0.09	3.64 ± 0.03	0.07 ± 0.010
	S27	0	6.55 ± 0.06	0	0	0	0	0
		6	5.62 ± 0.03	0.84 ± 0.03	0.90 ± 0.02	1.74 ± 0.05	3.50 ± 0.04	0.09 ± 0.021
		12	5.43 ± 0.00	1.44 ± 0.05	1.44 ± 0.04	2.87 ± 0.09	3.18 ± 0.07	0.09 ± 0.006
		24	5.13 ± 0.02	1.90 ± 0.03	1.40 ± 0.04	3.30 ± 0.07	4.26 ± 0.06	0.19 ± 0.015
		48	5.02 ± 0.02	1.95 ± 0.03	1.80 ± 0.04	3.75 ± 0.07	4.72 ± 0.06	0.19 ± 0.015
XOS	S26	0	6.52± 0.07	0	0	0	0	0
		6	5.63 ± 0.03	1.99 ± 0.07	1.39 ± 0.03	3.39 ± 0.10	6.46 ± 0.06	0.09 ± 0.012
		12	5.60 ± 0.02	2.03 ± 0.04	1.48 ± 0.01	3.51 ± 0.05	6.12 ± 0.08	0.10 ± 0.006
		24	5.16 ± 0.02	2.20± 0.04	1.54 ± 0.01	3.74 ± 0.05	6.62 ± 0.08	0.11 ± 0.006
		48	4.91 ± 0.02	2.28± 0.04	1.72 ± 0.01	4.00 ± 0.05	6.62 ± 0.08	0.11 ± 0.006
	S16	0	6.52± 0.04	0	0	0	0	0
		6	5.59 ± 0.02	2.37 ± 0.11	1.69 ± 0.06	4.06 ± 0.17	4.68 ± 0.10	0.06 ± 0.012
		12	5.25 ± 0.02	2.63 ± 0.10	1.77 ± 0.07	4.40 ± 0.17	4.80 ± 0.10	0.06 ± 0.012
		24	5.02 ± 0.02	2.76 ± 0.10	1.96 ± 0.07	4.72 ± 0.17	5.18 ± 0.10	0.07 ± 0.012
		48	5.00 ± 0.02	2.80 ± 0.10	1.96 ± 0.07	4.76 ± 0.17	5.20 ± 0.10	0.07 ± 0.012
	S27	0	6.52± 0.06	0	0	0	0	0
		6	5.57 ± 0.01	1.31 ± 0.04	2.42 ± 0.05	3.73 ± 0.09	6.82 ± 0.05	0.02 ± 0.010
		12	5.48 ± 0.00	1.44 ± 0.05	1.44 ± 0.04	2.87 ± 0.09	7.78 ± 0.07	0.03 ± 0.006
		24	4.98 ± 0.00	1.84 ± 0.05	1.90 ± 0.04	3.74 ± 0.09	7.78 ± 0.07	0.6 ± 0.006
		48	4.79 ± 0.00	1.98 ± 0.05	2.24 ± 0.04	4.22 ± 0.09	7.89 ± 0.07	0.7 ± 0.006

**Table 7:** Phospholipid composition of plasma membranes isolated from the livers of XOS-fed rats.

Lipids	Control (mol%)	XOS (mol%)
Lysophosphatidylcholine	2.3	1.1 *
Sphingomyeline	10.5	11.2 *
Phosphatidylcholine	39.6	41.7 *
Phosphatidylserine	9.4	9.3
Phosphatidylinositol	10.7	9.8
Phosphatidylethanolamine	27.5	26.9
Cholesterol/phospholipids	0.332	0.285 *

* *p* < 0.01. Results are the means of three separate experiments.

**Table 8:** Fatty acid composition of phosphatidylcholine in plasma membranes isolated from rat livers after intake of XOSs.

Fatty Acids	Control	XOS (mol%)
C16:0	27.5	28.1 *
C18:0	21.8	21.5
C18:1	12.2	12.8
C18:2	12.6	12.2
C20:4	23.7	21.8
C22:6	2.2	3.6

* *p* < 0.001. Values are the means of three separate experiments.
